# Chitosan-Based Schiff Bases (CSBs) and Their Metal Complexes: Promising Antimicrobial Agents

**DOI:** 10.3390/molecules30020207

**Published:** 2025-01-07

**Authors:** Domenico Iacopetta, Alessia Catalano, Jessica Ceramella, Annaluisa Mariconda, Assunta D’Amato, Paola Checconi, Stefano Aquaro, Pasquale Longo, Maria Stefania Sinicropi

**Affiliations:** 1 Department of Pharmacy, Health and Nutritional Sciences, University of Calabria, 87036 Rende, Italy; domenico.iacopetta@unical.it (D.I.); jessica.ceramella@unical.it (J.C.); s.sinicropi@unical.it (M.S.S.); 2 Department of Pharmacy-Drug Sciences, University of Bari “Aldo Moro”, Via Orabona, 4, 70126 Bari, Italy; 3 Department of Basic and Applied Sciences, University of Basilicata, Via dell’Ateneo Lucano, 10, 85100 Potenza, Italy; annaluisa.mariconda@unibas.it; 4 Department of Chemistry and Biology “A. Zambelli”, University of Salerno, Via Giovanni Paolo II, 132, 84084 Fisciano, Italy; asdamato@unisa.it (A.D.); plongo@unisa.it (P.L.); 5 Department for the Promotion of Human Sciences and Quality of Life, San Raffaele University, Via di Val Cannuta 247, 00166 Rome, Italy; paola.checconi@uniroma5.it; 6 Laboratory of Microbiology, IRCCS San Raffaele Roma, Via di Val Cannuta 247, 00166 Rome, Italy; 7 Department of Life, Health and Environmental Sciences, University of L’Aquila, Piazzale Salvatore Tommasi, 1, Blocco 11, Coppito, 67010 L’Aquila, Italy; stefano.aquaro@univaq.it

**Keywords:** Schiff bases, chitosan, antimicrobial, antibacterial, antifungal, metal complexes

## Abstract

The scientific interest in the chemical modification of chitosan to increase its solubility and application has led to its conjugation with Schiff bases, which are interesting scaffolds endowed with diverse biological properties. The resultant chitosan-based Schiff bases (CSBs) are widely studied in scientific literature due to the myriad of activities exerted, both catalytic and biological, including anticancer, anti-inflammatory, antioxidant, and especially antimicrobial ones. Antimicrobial resistance (AMR) is one of the major public health challenges of the twenty-first century because it represents a threat to the prevention and treatment of a growing number of bacterial, parasitic, viral, and fungal infections that are no longer treatable with the available drugs. Thus, in this review, we present a brief outline of the biological activities of CSBs as well as their complexes with metals, with a particular focus on the recent literature regarding the antimicrobial effect of these captivating derivatives.

## 1. Introduction

**Figure 1:**
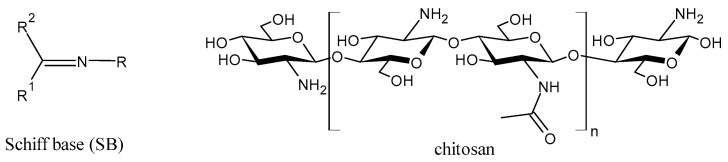
Structures of SBs and chitosan.

Chitosan-based Schiff bases (CSBs) are valuable organic compounds that are simply prepared by facilitating the reactions of the reactive amino groups of chitosan with different aldehydes/ketones. Specifically, Schiff base (SB) or imine or azomethine is a derivative of aldehydes or ketones in which the carbonyl group (C=O) has been replaced by an imine or azomethine group (Figure 1). SBs are considered privileged ligands [1] on account of their easy synthesis and because they are involved, as precursors and intermediates, in the synthesis of biologically active agents. In addition, several biological activities have been described for these compounds, including antiviral [2], antidiabetic [3], antihyperlipidemic [4], anti-inflammatory [5], anticancer [6], antimicrobial [7,8], and antioxidant. Combined with chitosan, crossing the blood–brain barrier, they could represent an innovative drug delivery system for neurodegenerative conditions such as Alzheimer’s, Parkinson’s, and Huntington’s diseases [9]. Chitosan is a biodegradable, biocompatible, non-toxic, and renewable biopolymer [10,11]. It is the principal derivative of chitin, which is the second most abundant natural polysaccharide on Earth after cellulose in terms of availability. The linear polysaccharide chitosan is a chiral compound [12] consisting of D-glucosamine and *N*-acetyl-D-glucosamine units connected by linear β-(1,4)-links and is usually obtained by deacetylation through either chemical or enzymatic processes (Figure 1) [13]. It is endowed with unique intrinsic properties like mucoadhesion, biodegradability, and biocompatibility, leading to the particular significance of this compound [14].

Chitosan has demonstrated diverse biological activities, such as antimicrobial and antiviral. In particular, it has also been reported to act as an antibacterial agent against resistant bacteria [15] and *Helicobacter pylori*, which is one of the most common bacterial infections worldwide and among the main etiological factors responsible for chronic gastritis, peptic ulcer disease, and stomach neoplasms [16,17], even in its multidrug-resistant form [18]. Moreover, chitosan is a broad-spectrum fungicide [19,20,21,22] that is also useful in food preservation [23,24]. The antiviral activity of chitosan is significantly increased by substitutions at the amino and hydroxyl groups; in fact, some substituted derivatives acquire antiviral efficacy against a broad spectrum of bacteriophage, plant, animal, and human viruses [25]. In vitro and in vivo studies evidenced that chitosan effectively stimulates cell adhesion, proliferation, and differentiation and has been suggested as a tool for tissue engineering and rehabilitation [26]. Under acidic conditions, chitosan undergoes protonation, resulting in the formation of positively charged amino groups (-NH_4_^+^), which may interact with the negative charges present on the microbial cell membrane through electrostatic interactions [27]. Cationic chitosan derivatives are able to inactivate enveloped viruses, i.e., membrane-coated viruses, such as HIV-1 and SARS-CoV-2 [28] and other coronaviruses [29]. However, the activity of chitosan is severely limited by its solubility [30]. Thus, the reaction of the amino group of chitosan with different groups leads to diverse types of structural modifications that improve its solubility. Chitosan derivatives, containing common functional groups, including alkyl and acyl groups, SBs, quaternary ammonia, guanidines, and heterocyclic rings, have expanded the application field of chitosan, representing significative compounds in the field of medical materials and biomedical science [31,32]. Chitosan derivatives represent significative compounds in the field of medical materials and biomedical science, demonstrating antimicrobial activity [33], also against enteric bacteria [34] and orthopedic and vaginal infections [35,36], as well as antioxidant, antitumor, anti-HIV, anti-inflammatory, antihypertensive, and antidiabetic activity, and they have been used in the treatment of Alzheimer’s disease [31,32]. Specifically, CSBs have been demonstrated to generally exhibit low toxicity and better antimicrobial properties than bare chitosan [37], even against dangerous bacteria, such as those belonging to the ESKAPE group of bacteria (*Enterococcus faecium*, *Staphylococcus aureus*, *Klebsiella pneumoniae*, *Acinetobacter baumanii*, *Pseudomonas aeruginosa*, and *Enterobacter* species), which are dubbed as “superbugs” as they are responsible for the majority of nosocomial infections. Given the increasing global prevalence in recent decades of AMR, widely referred to as the “*Silent Pandemic*”, urgent action toward this problem is needed [38]. The design and synthesis of new compounds acting as antimicrobials are desirable. Thus, this review focuses on chitosan and CSBs, specifically on their antimicrobial activities, highlighting the most recent and interesting studies carried out in the last two years in this field.

## 2. Chitosan as an Antimicrobial

Chitosan is a versatile biomacromolecule found abundantly in nature. It is obtained by removing part of the acetyl groups (usually more than 60%) of chitin. The journey of chitosan began in 1859. Since its first citation, on 9 December 1964, in the ScienceDirect database, it has received more than 30,000 citations [11,39]. In the 1990s, it entered the market in the United States under the category of dietary supplements. Due to its properties and the myriad of activities in biomedical applications, chitosan has been recently defined as an excellent biomacromolecule [31,40]. The antimicrobial activity of chitosan against bacteria and fungi is strongly affected by many factors, such as the type of microorganism, pH value, molecular weight (MW), degree of deacetylation (DD), and pattern of acetylation (PA) [41,42,43]. Chitosan can be classified into three different types: high molecular weight chitosan (HMWC, more than 700 kDa), medium molecular weight chitosan (MMWC, 150–700 kDa), and low molecular weight chitosan (LMWC, less than 150 kDa) [44]. Román-Doval suggested that both HMWC and MMWC have a preferential effect on Gram-positive bacteria due to their cell wall components but have no effect on Gram-negative bacteria, whereas LMWC inhibits Gram-negative bacterial growth [45]. On the other hand, DD plays an important role in determining chitosan bioactivities, as chitosan with a higher level of deacetylation can carry more positive charges through protonation, allowing it to adhere firmly to the surface of bacteria and have a better antibacterial effect [46]. Its usefulness in combination with other antifungals has been proposed to reduce drug resistance against human pathogenic or opportunistic fungi, as it has been recently shown to reduce caspofungin resistance in different drug-resistant *Candida* spp. [47]. Many drug delivery systems such as films, fibers, gels, nanoparticles, microparticles, liposomes, and injectable systems made of chitosan and other polysaccharides are often used [48,49]. Polysaccharide-based systems with chitosan have demonstrated interesting activities for dental drug delivery in the treatment of various diseases, including dental caries, periodontal disease, and endodontic disease [50], as well as in the plant agricultural field [51]. The antimicrobial and antioxidant activities of chitosan have also led to the use of chitosan-based coatings and films for food packaging [52]. Some chitosan nanoparticles obtained by using tea (*Camellia sinensis*) extract have shown interesting antimicrobial properties against the most devastating pathogens of rice viz., *Pyricularia grisea*, *Xanthomonas oryzae* in vitro [53]. Interesting results were obtained with chitosan-fabricated tellurium nanoparticles that exhibited antibacterial and antibiofilm activity on Gram-negative bacteria and also significant free radical scavenging activity against ABTS and DPPH free radicals and cytotoxicity against cancerous cells [54]. Other chitosan-based nanoparticles have been suggested as an alternative to sodium hypochlorite against *Enterococcus faecalis* and demonstrated higher antibacterial activity than chlorhexidine [55]. Moreover, the use of one-step spraying of protein-anchored chitosan oligosaccharide has been suggested as an antimicrobial coating for the preservation of food as a potential economic alternative to current commercial antimicrobial coatings [56]. Recently, chitosan hydrochloride and some chito-oligosaccharides and chito-oligogalacturonides demonstrated antimicrobial activity against phytopathogenic fungi and *E. coli*, thus suggesting its use instead of synthetic pesticides [57]. New studies have addressed the preparation of hydrogels containing chitosan for various activities [58], including bone and cartilage regeneration [59,60], wound healing [61], and disinfection [62]. The exact mechanism of the antibacterial action of chitosan has not been completely defined [63]. Multiple independent factors are likely involved. The first proposed mechanism is that chitosan may adhere to the negative charges of bacterial wall cells, initiating cell disruption and altering membrane permeability. The following attachment to DNA inhibits DNA replication, ultimately leading to cell demise. Another mechanism is that chitosan can inhibit microbial proliferation by selectively binding to trace metal elements acting as a chelating agent. The antibacterial activity of chitosan is influenced by pH. At low pH values, the electrostatic interaction between the positive charges of chitosan and the negative charges of the bacterial surface is essential for the activity: the higher the charge density, the greater the antibacterial activity. In addition, the antibacterial activity may be further increased by the presence of additional amino groups on the chitosan backbone. Finally, the mode of action of chitosan particles may be influenced by their size and shape, with bigger particles being incorporated into the cell surface and altering cell permeability [63]. The antimicrobial activity of chitosan can be further enhanced by its complexation with different metals, as shown by Brunel et al. (2013) [64], who reported a strong synergistic effect between chitosan and copper in inhibiting the growth of Fusarium graminearum, the fungal pathogen that causes head blight in cereals. Other studies have been carried out against bacteria [65,66], including *H. pylori* [67] and multidrug-resistant bacteria [68,69]. Good antibacterial activities have been demonstrated by complexes with silver [70,71] as well as selenium [72,73], copper [74], gold [75], and iron [76,77]. Chitosan-modified molybdenum selenides have recently demonstrated activity against *H. pylori* [78]. Kritchenkov et al. (2020) [79] reported the synthesis and biological activity evaluation as antimicrobial of zinc(II)/chitosan-based composites. One compound showed higher antibacterial activity in vitro against *E. coli* and *S. aureus* than chitosan as well as the references ampicillin and gentamicin, and was non-toxic. The mechanism was suggested to be related to a symbiotic effect of increased cationic zinc complex with chitosan compared with the starting chitosan and the presence of zinc(II) in the polymeric matrix of the composite.

## 3. Chitosan-Based Schiff Bases (CSBs)

### 3.1. Synthetic Routes to Obtain CSBs

CSBs are typically synthesized by condensation of chitosan’s amino groups with the carbonyl groups of aldehydes/ketones via the elimination of water molecules. The first CSB was described by Hirano et al. in 1977 and was obtained by reacting chitosan with different aldehydes using the acetic acid–methanol solvent mixture [80]. Then, acetic acid, ethanol, methanol, or their mixtures were used as solvents, either at ambient or refluxing temperature conditions. In addition, some reports describe the use of DMF, water, and ionic liquids for the synthesis of these compounds. In some cases, the synthesis of CSBs was obtained with a different route: the amino and hydroxyl groups of chitosan were protected by coordination with copper and the –CH_2_OH groups were oxidized into formyl groups, which can in turn react with different amines for the preparation of CSBs [81]. Other recently reported synthetic routes are described below.

### 3.2. Chemical and Biological Activities of CSBs

CSBs have been studied for several biological applications [82], such as antimicrobial applications [83,84,85,86]; anticancer applications for colon cancer [87], breast cancer [88], esophageal cancer [89], and melanoma [90]; as protective therapy for cisplatin-induced hepatotoxicity [91]; as an antioxidant [92,93]; for UV-protective applications [94]; and as a drug carrier [95,96] owing to their unique intrinsic properties like mucoadhesion, biodegradability, and biocompatibility [14,97]. In addition, CSBs can be considered very promising materials for industrial applications such as wastewater treatment. The efficient removal, by using CSBs, of toxic substances from aqueous media has been described, including emerging contaminants, such as polycyclic aromatic hydrocarbons, pharmaceutical and personal care products [98,99,100], and some of the most noxious dyes, including Congo red [101], Malachite green [102], Bismarck brown R, and Rhodamine B [103]. In addition, the use of CSBs for the elimination of metals from wastewater including Cu^2+^, Zn^2+^, Fe^3+^, and the most dangerous Pb^2+^, Cd^2+^, Hg^2+^, and Cr^6+^ has been widely reported [104,105,106,107,108,109,110,111,112]. Moreover, the detection of catecholamines/antibiotics and removal of antibacterial and antifungal treatments against phytopathogens have been reported [113,114,115,116]. In addition, the usefulness of CSBs as catalysts [117,118,119,120,121], corrosion inhibitors [122,123,124,125,126], charring agents [127], sensors [128,129,130], and for the preparation of smart hydrogels [131,132] has been outlined. Finally, given the well-known usefulness in various fields of complexes of SBs with transition metals [32,133,134,135,136,137,138], recent studies have addressed the use of CSBs complexed with metals (e.g., Fe, Cu, Ni, Pd, Pt, and Zn) as catalysts [118,139,140], antimicrobial [141,142] and antitumor agents [143], and also in the form of hydrogels [66,144,145,146]. Different synthetic routes have been reported for the preparation of CSBs. The most recent explorations are directed towards green synthetic routes by using non-conventional green methods such as microwave irradiation, green solvent, ultrasound irradiation, and one-pot synthesis, which have been recently described in the literature [147,148].

### 3.3. Chitosan-Based Schiff Bases (CSBs) as Antimicrobials

In this paragraph, the most recent articles regarding the antimicrobial activities of CSBs are summarized (Table 1). Antimicrobial activity evaluation was obtained via the agar diffusion method unless otherwise indicated. The minimum inhibitory concentration (MIC) (the lowest concentration that resulted in maintenance or reduction in inoculum viability) and minimum bactericidal concentration (MBC) (the least concentration of antimicrobial agent required to kill microorganisms) are provided when reported, otherwise, the inhibitory zone diameter (IZD) is given. MIC, MBC, and IZD values for standards and/or chitosan are reported in the text. Studies were carried out against Gram-positive bacteria (*Staphylococcus aureus*, *Staphylococcus haemolyticus*, *Staphylococcus epidermidis*, *Bacillus subtilis*, and *Listeria innocua*), Gram-negative bacteria (*Escherichia coli*, *Pseudomonas aeruginosa*, *Klebsiella pneumoniae*, and *H. pylori*), and fungi (*Candida albicans*, *Botrytis cinerea,* and *F. graminearum*). Some of the bacteria studied belong to the ESKAPE group of bacteria [149].

Tamer et al. (2024) [150] reported a study on a CSB bearing a phenolic group (**Va.Ch.SB**) prepared by a click reaction between chitosan and vanillylidene acetone (also known as feruloylmethane or dehydrozingerone, a phenolic compound derived from ginger) for its antimicrobial and antioxidant properties. Antimicrobial activity evaluation was carried out against Gram-positive (*S. aureus* MT1 (KY421197) and *S. haemolyticus* MST1 (KY550377)) and Gram-negative (*E. coli* MST4 (KY550380), *P. aeruginosa* MST2 (KY550378), and *K. pneumoniae* MST3 (KY550379)) bacteria isolated from various wound types. The comparison was carried out with chitosan. Except for *K. pneumoniae*, the antibacterial activity was higher for the CSB than that found for chitosan (IZD = 29.65 ± 0.562 mm, 23.58 ± 1.078 mm, 23.58 ± 0.234 mm, 30.69 ± 0.646 mm, and 22.27 ± 0.552 mm against *E. coli*, *S. haemolyticus*, *P. aeruginosa*, *K. pneumoniae*, and *S. aureus*, respectively). The authors suggest that the inclusion of phenolic groups determined the enhancement of antibacterial activity, due to the increase in the hydrophobic–hydrophobic interaction between the polymer and the cell wall peptidoglycan, such as in Gram-negative bacteria.

Hamed et al. (2024) [151] reported a study on two CSB (**Cs-SBA** and **Cs-SBBr**) nanoparticles tested for their antimicrobial activity against *H. pylori* ATCC 700392 and the inhibitory potential of cyclooxygenases (COX-1 and COX-2). They were obtained by condensation reaction of chitosan with 2-(4-formylphenoxy)-*N*-phenylacetamide and *N*-(4-bromophenyl)-2-(4-formylphenoxy) acetamide in ethanol. Nanoparticles were obtained using the ionic gelation method. The antimicrobial activities of the CSB nanoparticles were higher than those of chitosan nanoparticles (IZD, MIC, and MBC values: 21.83 ± 0.29 mm, 31.25 ± 0.03 µg/mL, and 31.25 ± 0.04 µg/mL, respectively) and the positive control (mixture of amoxicillin 0.05 mg/mL + clarithromycin 0.05 mg/mL + metronidazole 0.8 mg/mL; IZD, MIC, and MBC values: 20.0 ± 0.50 mm, 15.62 ± 0.07 µg/mL, and 15.62 ± 0.05 µg/mL, respectively). Interestingly, **Cs-SBBr** nanoparticles also demonstrated COX enzyme inhibition activity against COX-2 that was higher than indomethacin and celecoxib, and no pronounced cytotoxic effect was found against Vero cells CCL-81, as determined by MTT assay. Given the high risk of heart attack and stroke that caused rofecoxib and other highly selective COX-2 inhibitors to be retired from the market, and the side effects related to the use of celecoxib and indomethacin [152,153], the authors suggested these nanoparticles as an alternative to cure *H. pylori* and prevent gastric cancer.

Moustafa et al. (2024) [154] studied several quaternized salicylidene CSBs as antibacterial and antibiofilm agents against *P. aeruginosa*, *K. pneumoniae*, *E. coli,* and *Bacillus subtilis*. The protocol for the synthesis was simple, safe, and inexpensive and used multiple chemical approaches such as chloromethylation, quaternization, catalytic reduction, and nucleophilic substitution, starting from paraformaldehyde. The values of antibacterial activity are expressed as mean inhibitory zone diameters (in mm). The control (chloramphenicol) group showed an inhibition zone of 15 ± 0.75 mm for *P. aeruginosa*, 14 ± 0.84 mm for *K. pneumoniae*, 13 ± 0.46 mm for *E. coli,* and 13 ± 0.65 mm for *B. subtilis.* **QSCSB2** was the most active, followed by **QSCSB1** as an antibacterial. Regarding the activity against *P. aeruginosa* biofilm, **QSCSB1** demonstrated moderate antibiofilm activity, with an inhibition percentage of 51.35%, while **QSCSB2** displayed the highest antibiofilm activity, leading to the inhibition of biofilm formation by 64.86%.

Lee et al. (2024) [155] described an injectable, chitosan-based hydrogel (**SC/PNF** hydrogel) prepared by SB reaction of the aldehyde groups on poly(*N*-isopropylacrylamide)-*co*-2-(4-formylbenzoyloxy)ethyl methacrylate [Poly(NIPAM-co-FBEMA)] and the amino groups of chitosan and studied its antibacterial and sustained release application. The MTT assay run on L929 fibroblasts showed good biocompatibility for the pristine **SC/PNF** hydrogel, demonstrating a significant increase in cell proliferation; the hydrogel was also demonstrated to be non-toxic, non-irritating, and non-allergenic. Moreover, the agar diffusion method showed that vancomycin-wrapped **SC/PNF** hydrogel manifested excellent antibacterial activity against Gram-positive bacteria (*S. aureus*), showing a rapid bacterial-killing effect with a clear inhibition zone that varied depending on the molar percent of FBEMA content (10 or 5). It is noteworthy that **SC/PNF** hydrogels (without vancomycin) exhibit a loosened gel structure leading to liquefaction while incubated with *S. aureus* at 37 °C for 24 h. This could be attributed to the outstanding biocompatibility of chitosan-based hydrogel, which was digested by the bacteria.

Cui et al. (2024) [156] reported the synthesis, antimicrobial and antioxidant activities, as well as molecular docking studies, of chitosan derivatives containing glycine SBs as potential succinate dehydrogenase inhibitors. The synthesis was obtained by the ethylcarbodiimide hydrochloride (EDCI)/*N*-hydroxysuccinimide (NHS) coupling reaction, by dissolving the SB in morpholinoethanesulphonic acid buffer. The antibacterial and antifungal activities were studied against two species of bacteria (*S. aureus* and *E. coli*) by the broth microdilution method, and two species of plant-pathogenic fungi (*B. cinerea* and *F. graminearum*) by the mycelia growth rate method. Some of the studied chitosan derivatives (**CSGDBH** and **CSGDCH**) displayed high antibacterial and antifungal activities, with **CSGDCH** being the most active (chitosan was used as a reference for antibacterial studies: MIC and MBC > 16 mg/mL; the fungicide carbendazim showed 100% inhibitory index at 0.1 mg/mL against both fungi).

Muñoz-Nuñez et al. (2023) [157] studied a thiazolium chitosan derivative (**CSMTBAQ**) that demonstrated higher water solubility and excellent antimicrobial properties, which were higher than chitosan. For the synthesis, 4-(2-(4-methylthiazol-5-yl)ethoxy)-4-oxobutanoic acid (MTBA) was quaternized into MTBAQ-bearing cationic thiazolium groups. It was then incorporated into chitosan through selective *N*-acylation of the amino group by using EDC/NHS to create a more active ester. The antimicrobial assays were run on bacteria (*L. innocua*, *S. epidermidis*, *S. aureus*, and *S. aureus* resistant to methicillin (MRSA), and *E. coli*) and the yeast *C. albicans*. The best results were obtained against *L. innocua* and *S. epidermidis.* Unmodified chitosan was reported as a reference typically showing MIC values higher than 256 µg/mL against Gram-positive and Gram-negative bacteria [158].

Abdel-Baky et al. (2023) [159] described a chitosan–quinoline SB (**CHQ 1.0**) and its antibacterial activity evaluation against *S. haemolyticus* and *E. coli*. For the synthesis, the quinoline derivative, dissolved in ethanol, was added to chitosan dissolved in acetic acid under stirring at room temperature. The new compound demonstrated higher antibacterial activity than chitosan against both bacteria (chitosan: IZD = 33.5 ± 0.23 mm and 28 ± 0.42 mm, against *S. haemolyticus* and *E. coli*, respectively). Interestingly, this compound also showed antidiabetic activity higher than chitosan (acarbose and berberine were used as standards), probably through the inhibition of α-amylase and α-glucosidase enzymes.

Pawariya et al. (2024) [103] described a CSB, namely **CCS**, for the removal of pernicious dyes, specifically Bismarck brown R and Rhodamine B, from wastewater and their activity as antibacterials against *E. coli*, *P. aeruginosa*, *S. aureus*, and *B. subtilis*. For the synthesis, benzaldehyde dissolved in methanol was added to chitosan dissolved in acetic acid (to which methanol had also been added) under stirring at room temperature. Compound **CCS** showed interesting antibacterial properties against bacteria (chitosan data for comparison were not given). The percentages of the removal of dyes were 93 to 99%.

Zhang et al. (2023) [160] reported a study on pyridine-4-aldehyde grafted onto chloracetyl chitosan oligosaccharide, bearing additional positive charges (**BPCACS**, **2CBPCACS**, **3CBPCACS**, and **4CBPCACS**) studied as antibacterials against *S. aureus* and *E. coli* and as antioxidants. For the synthesis, chitosan was dissolved in water at room temperature and then chloracetyl chloride was added. The final compounds were obtained by dissolving the obtained chloracetyl chitosan oligosaccharide and SBs of pyridine-4-aldehyde in DMF. All the compounds were more active than chitosan (chitosan: MIC and MBC > 16 mg/mL against *S. aureus* and *E. coli*). **BPCACS** was the most active of the series both as an antibacterial and antioxidant.

**Table 1:** Antimicrobial activities of CSBs.

Structure	Compound	Antimicrobial Activity	Ref.
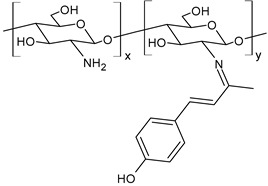	**Va.Ch.SB**	IZD = 34.60 ± 0.343 mm (*E. coli*) IZD = 25.53 ± 1.083 mm (*S. haemolyticus*) IZD = 26.05 ± 0.269 mm (*P. aeruginosa*) IZD = 28.54 ± 0.963 mm (*K. pneumoniae*) IZD = 24.98 ± 0.280 mm (*S. aureus*)	[150]
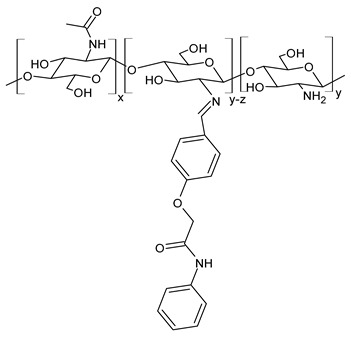	**Cs-SBA (NPs)**	IZD = 23.33 ± 0.58 mm (*H. pylori* ATCC 700392) MIC = 15.62 ± 0.05 µg/mL (*H. pylori* ATCC 700392) MBC = 15.62 ± 0.50 µg/mL (*H. pylori* ATCC 700392)	[151]
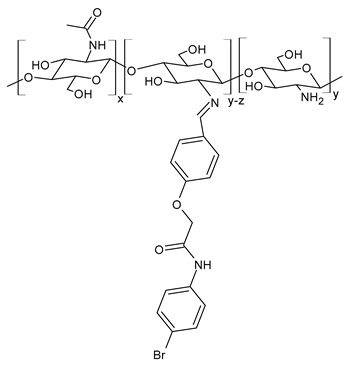	**Cs-SBBr (NPs)**	IZD = 27.00 ± 0.41 mm (*H. pylori* ATCC 700392) MIC = 3.90 ± 0.03 µg/mL (*H. pylori* ATCC 700392) MBC = 3.90 ± 0.20 µg/mL (*H. pylori* ATCC 700392)	[151]
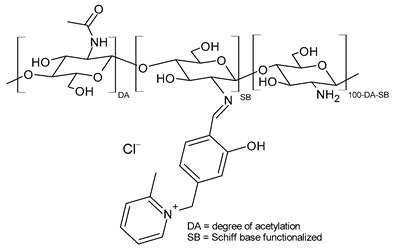	**QSCSB1**	IZD = 20 ± 1 mm (*P. aeruginosa* ATCC 9027) IZD ~ 18 mm (*K. pneumoniae* ATCC 13883) IZD ~ 18 mm (*E. coli* ATCC 19404) IZD ~ 14 mm (*B. subtilis* ATCC 6633) MIC = 250 ± 12.5 μg/mL (*P. aeruginosa* ATCC 9027) Percentage inhibition = 51.35% (against *P. aeruginosa*-induced biofilm)	[154]
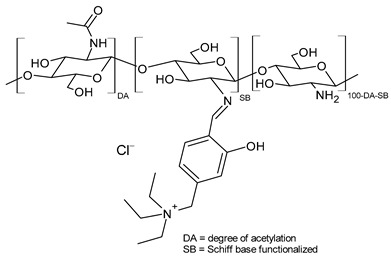	**QSCSB2**	IZD = 23 ± 1.15 mm (*P. aeruginosa* ATCC 9027) IZD = 23 ± 1.38 mm (*K. pneumoniae* ATCC 13883) IZD = 20 ± 0.7 mm (*E. coli* ATCC 19404) IZD = 18 ± 0.9 mm (*B. subtilis* ATCC 6633) MIC = 125 ± 7.5 μg/mL (*P. aeruginosa* ATCC 9027) Percentage inhibition = 64.86% (against *P. aeruginosa*-induced biofilm)	[154]
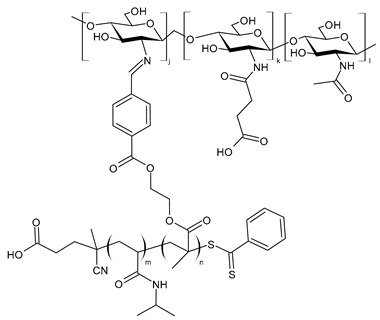	**SC/PNF (hydrogel)**	IZD = 1.85 cm (for SC/PNF_10__7.5 wt% with vancomycin against *S. aureus*) IZD = 2.05 cm (for SC/PNF_10__5 wt% with vancomycin against *S. aureus*) IZD = 1.91 cm (for SC/PNF_5__7.5 wt% with vancomycin against *S. aureus*)	[155]
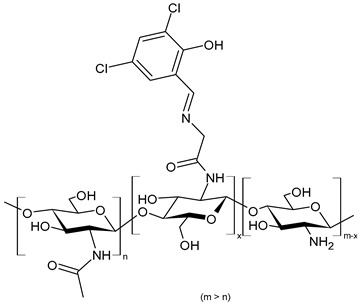	**CSGDCH**	MIC = 0.03125 mg/mL (*E. coli*) MIC = 0.0156 mg/mL (*S. aureus*) MBC = 0.03125 mg/mL (*E. coli*) MBC = 0.0156 mg/mL (*S. aureus*) Inhibitory index (%) = 100% (at 1.0 mg/mL); 79.34% (at 0.1 mg/mL (*B. cinerea*) Inhibitory index (%) = 100% (at 0.5 mg/mL); 96.87% (at 0.1 mg/mL (*F. graminearum*)	[156]
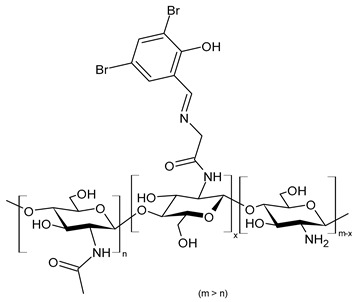	**CSGDBH**	MIC = 0.03125 mg/mL (*E. coli*) MIC = 0.0156 mg/mL (*S. aureus*) MBC = 0.03125 mg/mL (*E. coli*) MBC = 0.0156 mg/mL (*S. aureus*) Inhibitory index (%) = 98.52% (at 1.0 mg/mL (*B. cinerea*) Inhibitory index (%) = 100% (at 0.5 mg/mL) (*F. graminearum*)	[156]
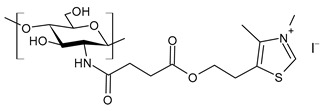	**CSMTBAQ**	MIC = 8 µg/mL (*L. innocua* ATCC 33090) MIC = 8 µg/mL (*S. epidermidis* ATCC 12228) MIC = 31 µg/mL (*S. aureus* ATCC 29213) MIC = 31 µg/mL (MRSA ATCC 43300) MIC = 250 µg/mL (*C. albicans* ATCC 200955) MIC > 1000 µg/mL (*E. coli* ATCC 25922)	[157]
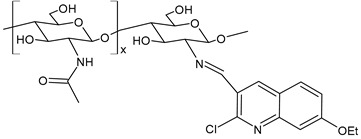	**CHQ (1.0)**	IZD = 37.0 ± 0.45 mm (*S. haemolyticus*) IZD = 32.5 ± 0.37 mm (*E. coli*)	[158]
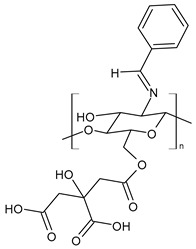	**CCS**	IZD = 13.5 ± 0.70 mm (*E. coli* MTCC 1687) IZD = 10.4 ± 0.19 mm (*P. aeruginosa* MTCC 741) IZD = 13.7 ± 0.13 mm (*S. aureus* MTCC 902) IZD = 11 ± 0.91 mm (*B. subtilis* MTCC 441)	[103]
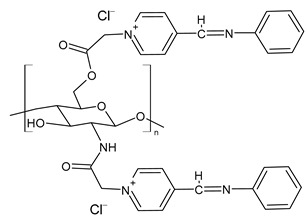	**BPCACS**	MIC = 0.125 mg/mL (*S. aureus*) MBC = 0.25 mg/mL (*S. aureus*) MIC = 0.25 mg/mL (*E. coli*) MBC = 0.5 mg/mL (*E. coli*)	[160]
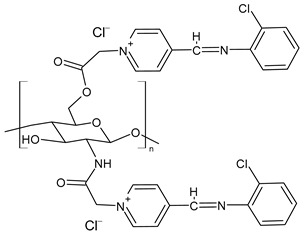	**2BPCACS**	MIC = 0.5 mg/mL (*S. aureus*) MBC = 1 mg/mL (*S. aureus*) MIC = 1 mg/mL (*E. coli*) MBC = 2 mg/mL (*E. coli*)	[160]
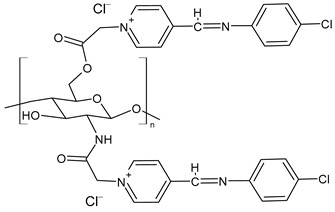	**3BPCACS**	MIC = 1 mg/mL (*S. aureus*) MBC = 2 mg/mL (*S. aureus*) MIC = 1 mg/mL (*E. coli*) MBC = 2 mg/mL (*E. coli*)	[160]
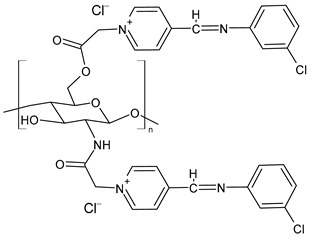	**4BPCACS**	MIC = 1 mg/mL (*S. aureus*) MBC = 2 mg/mL (*S. aureus*) MIC = 2 mg/mL (*E. coli*) MBC = 2 mg/mL (*E. coli*)	[160]

### 3.4. Chitosan-Based Schiff Bases (CSBs) Complexed with Metals as Antimicrobials

Complexation between modified chitosans, such as CSBs, and various metals has been promoted to obtain more stable compounds than those prepared with unmodified chitosan [161]. This paragraph regards the most recent studies of CSBs complexed with metals showing higher activity than CSBs. CSB complexes with metals can permeate the bacterial cell membrane efficiently and then easily prevent or inhibit bacterial growth. In addition, the metal ions could easily combine with oxygen in organisms and then denatured proteins or enzymes through the effect on the synthesis of amino acids, proteins, lipoproteins, and coenzymes according to the formation of stable complexes. In 2020, Barbosa et al. [162] reported a study on several complexes of CSBs with Zn(II), Pd(II), and Pt(II) against *Pseudomonas syringae* pv. *tomato*, which causes bacterial speck of tomato, and the fungal pathogen *F. graminearum*. The complexes exhibited significantly higher antibacterial efficiency against *Pseudomonas syringae* pv. *tomato*, indicating a different interaction between the complexes with Gram-negative bacteria and fungi. The chitosan–salicylaldehyde SB silver nanoparticles reported by Alharhi et al. (2022) [163] showed antibacterial activity against *E. coli* and *P. aeruginosa* higher than chitosan. The activity against the fungus *Penicillium notatum* was even higher than the reference polymixin B sulfate. The studies summarized below demonstrate the activity of CSBs complexed with metals against Gram-positive (*S. aureus*) and Gram-negative (*E. coli*, *P. aeruginosa,* and *H. pylori*) bacteria and fungi (*Pythium vexans* and *Phytophthora capsici*). The compounds were characterized by X-ray diffraction (XRD), energy-dispersive spectroscopy (EDS) analysis, transmission electron microscope (TEM), field emission scanning electron microscope (SEM), thermal gravimetric analysis (TGA), and Fourier transfer infrared spectroscopy (FT-IR) as detailed below.

**Figure 2:**
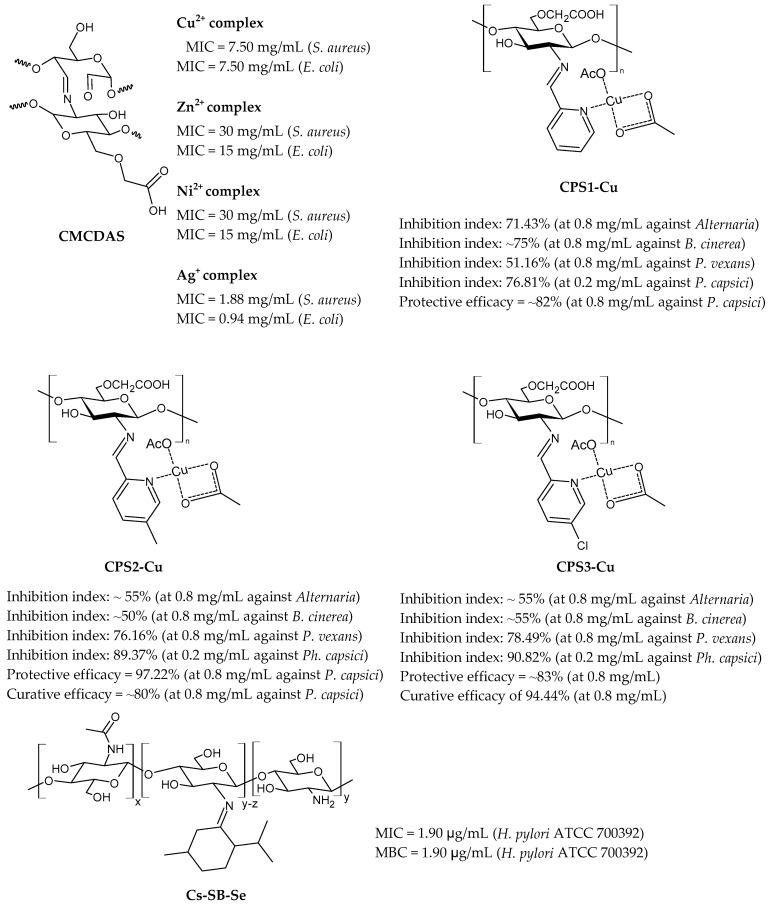
Structure of complexes from Refs. [164,165,166].

Ouyang et al. (2023) [164] reported a study on a chitosan–dialdehyde starch Schiff base (**CMCDAS**), with different nitrogen contents, and its metal complexes with copper, zinc, nickel, and silver (Figure 2). The compounds were synthesized by corn starch, sodium periodate, carboxymethyl chitosan, and metal ions. Characterization of products was generally obtained by XRD, SEM, EDS, TGA, and FT-IR. The antibacterial effect of carboxymethyl chitosan dialdehyde starch Schiff base increased with the increase in nitrogen content (against both bacteria, the MIC values were as follows: MIC > 120 mg/mL for N = 4.51%; MIC = 120 mg/mL for N = 4.85%l and MIC = 30 mg/mL for N = 5.71%). The antibacterial activities of **CMCDAS** against *S. aureus* and *E. coli* were enhanced by complexation with metals. Specifically, the most active compound was a complex with silver. The better antibacterial activity exerted by metal complexes against *E. coli* and *S. aureus* with respect to the carboxymethyl chitosan dialdehyde starch Schiff base was caused by the ligand orbital overlap and the charge sharing between donor groups and the positive charge of metal ions, according to the increasing penetrability of metal atoms in the microbial lipid membrane.

Liu et al. (2024) [165] reported a series of chitosan oligosaccharide complexes with copper-bearing pyridine moieties (**CPS1-Cu**, **CSP2-Cu**, and **CSP3-Cu**) to study the slow-release copper fungicide effect. Copper complexes were prepared via a sequential three-step process. In the first step, Schiff bases were prepared; then, the 6-OH group on chitosan was replaced by monochloroacetic acid; finally, the ligands obtained were reacted with copper acetate to form the desired copper complexes. The compounds were characterized by UV-Vis, ^1^H NMR and ^13^C NMR, FT-IR, and DFT calculations. Elemental analysis was used to evaluate the DD and degree of substitution. The in vitro and in vivo antifungal activities were evaluated against fungi generally affecting plants, specifically *Bacillus cinerea*, which causes the grey mold disease, *Alternaria*, a widely distributed fungus that causes leaf spot and black spot, *Pythium vexans*, which may cause significative losses in vegetables and fruit, and *Phytophthora capsici*, one of the most destructive pathogens of vegetables. In vitro studies were carried out using the mycelium growth rate method (mycelial radial growth was measured only when the negative control colony reached the edge of the plate). At the concentration of 0.4 mg/mL, all the complexes completely inhibited the growth of *P. vexans* and *P. capsici*, demonstrating more activity than thiodiazole copper and basic copper sulfate, which were used as positive controls. Moderate activity was shown against *Alternaria* and *B. cinerea*, with **CSP3-Cu** being more active than the other two. In vivo experiments were run against *P. capsici* on pepper seedlings to evaluate the protective and curative efficacy. The highest protective efficacy was shown by the **CPS2-Cu** complex, while the **CPS3-Cu** complex demonstrated the highest curative efficacy, which was even higher than the positive controls (thiodiazole copper: protective efficacy = 83.33% and curative efficacy = 69.44%; basic copper sulfate: protective efficacy = 86.11% and curative efficacy = 75.00%). The compounds were suggested as new biogreen copper fungicides.

Hamed et al. (2024) [166] described some chitosan menthone SB nanocomposites with silver, selenium, and iron. CSB was obtained by adding menthone to a solution of chitosan in acetic acid. The most interesting was the complex with selenium (**Cs-SB-Se**), which showed antibacterial activity against *H. pylori* ATCC 700392 that was much higher than the corresponding CSB alone and the control. Clarithromycin (0.05 mg/mL), metronidazole (0.8 mg/mL), and amoxicillin (0.05 mg/mL) as a mixture was used as control giving 15.62 μg/mL for both MIC and MBC; the complex was also more active than those previously reported by the same group [151]. Interestingly, **Cs-SB-Se** nanoparticles also showed anti-inflammatory activity higher than celecoxib and indomethacin against cyclooxygenases (mainly COX-1) and no pronounced cytotoxic effect against Vero cells CCL-81, as determined by MTT assay. Cs-SB nanoparticles loaded with metals were characterized by using X-ray diffraction on powder samples. Energy-dispersive spectroscopy analysis confirmed the existence of Ag, Se, and Fe; a transmission electron microscope was used to validate the size, morphological form, and distribution of the Schiff base nanocomposites. The stability behavior of Cs-SB-Se was determined by using zeta-potential measurement, which indicated its excellent stability by electrostatic repulsion forces.

**Figure 3:**
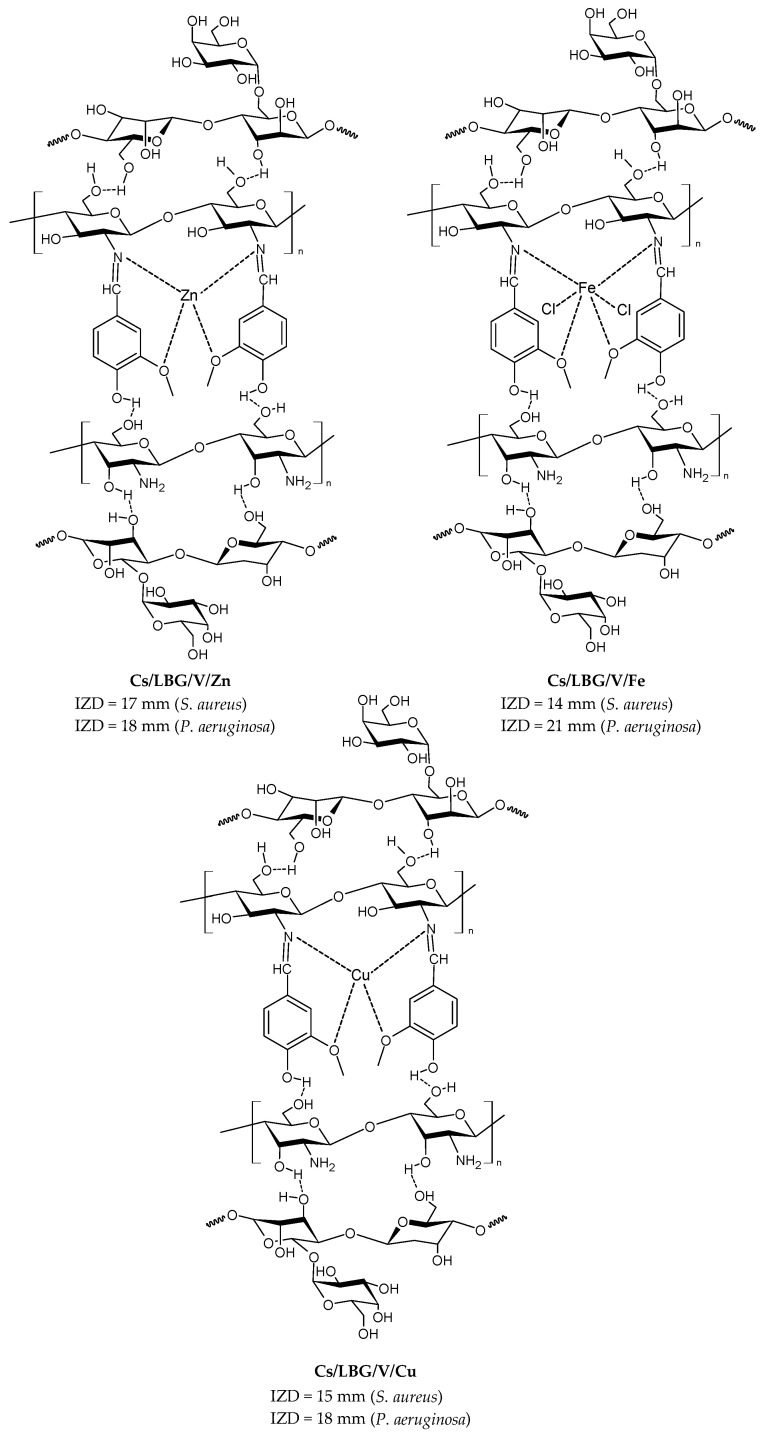
Structure of complexes from Ref. [167].

Matar et al. (2023) [167] studied some binary blended hydrogel films (**Cs/LBG/V/Zn**, **Cs/LBG/V/Fe**, and **Cs/LBG/V/Cu**; Figure 3) obtained by reaction of vanillin crosslinked to chitosan and locust bean gum (or carob gum), which is a galactomannan obtained from the seed endosperm of the carob tree, and their complexes with Fe(III), Zn(II), and Cu(II) for the antibacterial activity against *S. aureus* and *P. aeruginosa.* Rifampicin was used as a positive control (IZD = 14 mm and 25 mm against *S. aureus* and *P. aeruginosa*, respectively). All hydrogel films and their metal complexes exhibited good tensile strength and showed antibacterial activity against both bacteria. The mechanism of antibacterial activity in **Cs/LBG/V** hydrogels involves the release of metal ions (iron, zinc, and copper), which produce toxic effects on bacteria. These metal ions can disrupt bacterial metabolic processes leading to damage of the cell membrane and bacterial death. The hydrogels with chitosan were slightly more efficient than other hydrogels and antibiotics derived from different gums [168,169]. The authors used UV–Vis, FT-IR, XRD, SEM, EDX, and TGA analysis for the chemical characterization.

## 4. Conclusions

The high mortality rates associated with bacterial and fungal infections, as well as the growing number of multidrug-resistant strains, make the search for more effective antimicrobial therapies urgent. The coupling of chitosan, a semi-synthetic linear, biodegradable, non-toxic, and renewable polysaccharide, with aldehyde or ketones to obtain CSBs has been broadly used for boosting the antimicrobial activity of native chitosan; their synthesis is generally easy and not expensive. Interesting results were obtained for these compounds against a lot of bacteria and fungi, including the dangerous bacteria belonging to the ESKAPE group. An interesting and noteworthy inhibitory activity was found against *H. pylori*, along with the selective inhibition of COX-2. New studies are needed in this view, in order to find new drugs to cure *H. pylori* and prevent gastric cancer, as an alternative to rofecoxib and other highly selective COX-2 inhibitors. The latter have been withdrawn from the market for their negative effects on the cardiovascular system, and it is recommended to avoid the use of celecoxib and indomethacin. In addition, complexes of CSBs with metals have shown promising results as antimicrobials, which are generally better than CSBs alone. Interestingly, chitosan and its derivatives and complexes have shown a strong inhibitory effect against the growth of *F. graminearum*, the fungal pathogen that causes head blight in cereals and *Pseudomonas syringae* pv. *tomato*, which causes bacterial speck of tomato. CSB hydrogel films and their complexes with metals have demonstrated antimicrobial activity, thus representing interesting biomaterials for drug release, wound healing, and agriculture applications. CSBs copper complexes are also suggested as an effective approach to developing slow-release copper fungicides in green agriculture. CSBs have also demonstrated a good activity for the removal of pernicious dyes from wastewater. The research of chitosan and its derivatives, specifically CSBs, as antimicrobials with low toxicity is still ongoing. Researchers are exploring new methods and technologies to obtain these interesting compounds, including combinations with metals. Several in vitro studies have been carried out, obtaining good results. The antimicrobial activity of chitosan and its derivatives may be very advantageous for tissue engineering applications requiring infection control. However, the exact mechanism responsible for the antimicrobial activity has not been clearly defined. Future studies should deeply examine the interacting mechanism between these compounds and bacteria, as well as fungi. However, there is still a need to investigate the different properties and applications of these complexes, as well as to synthesize new complexes with additional applications. The data reported in this review emphasize that CSBs and their complexes with metals might be suggested as intriguing antimicrobial contenders to pure chitosan.

## Data Availability

Supporting data are available within the manuscript.
